# Sixteen Genome Sequences of Denitrifying Bacteria Assembled from Enriched Cultures of Anaerobic Pig Manure Digestate

**DOI:** 10.1128/MRA.00782-21

**Published:** 2021-09-30

**Authors:** Xinhui Wang, Baoyu Xiang, Menghui Zhang, Xiaojun Zhang

**Affiliations:** a State Key Laboratory of Microbial Metabolism, Shanghai Jiao Tong University, Shanghai, China; b Joint International Research Laboratory of Metabolic and Developmental Sciences, Shanghai Jiao Tong University, Shanghai, China; c School of Life Sciences and Biotechnology, Shanghai Jiao Tong University, Shanghai, China; Montana State University

## Abstract

We report 16 genomes assembled from the metagenome of pig manure digestate enriched with the addition of N_2_O. These denitrifying bacterial genomes all contain the *nosZ* gene, encoding N_2_O reductase. Their sizes range from 1,902,599 bp to 6,264,563 bp, with completeness of 75.03% to 98.89%, GC contents of 32.86% to 69.66%, and contamination of 0% to 8.4%.

## ANNOUNCEMENT

The greenhouse gas N_2_O is produced during nitrification and denitrification (1–4). The N_2_O reductase encoded by the *nosZ* gene is the only enzyme that can reduce N_2_O to the nongreenhouse gas N_2_ (5–8). In this study, bioinformatic tools were used for genome assembly and annotation of *nosZ* gene-containing bacterial genomes recovered from the metagenomic data for samples enriched with N_2_O.

The fresh digestate collected from an anaerobic tank for pig manure fermentation was incubated anaerobically for 1 week at 25°C with N_2_O, and the enriched digestate was then inoculated into gamma-ray-sterilized red soil and fluvo-aquic soil for another 1 week of anaerobic incubation; the incubated soil was inoculated into gamma-ray-sterilized digestate for another 1 week. After four rounds of soil-digestate reciprocal transfer and incubation with N_2_O, fresh digestate and N_2_O-enriched digestate, as well as the first and fourth rounds of incubated soil and digestate, were collected for DNA extraction with the Omega Bio-Tek soil DNA kit and library construction using the Illumina TruSeq DNA sample preparation guide. The metagenomes of 30 samples were paired-end sequenced on the Illumina NovaSeq platform. Finally, an average of 77,340,278 reads of 150 bp were obtained for each library.

Cutadapt (v1.17) (https://github.com/marcelm/cutadapt) was used to identify and cut off the adapter sequence. Fastp (v0.20.0) (https://github.com/OpenGene/fastp) was used to screen the quality of the sequence by the sliding window method. The sequences with lengths of less than 50 bp and those with fuzzy bases were removed to yield clean data.

MEGAHIT (v1.2.3-beta) (9) was used to assemble the contigs with the option of minimum contig length of 500. MetaBAT2 (v2.15) (10) was used for binning, and CheckM (v1.0.18) (11) was used to assess the completeness and contamination of the bins. Then, dRep (v2.5.4) (12) was used to remove the redundant bins, and 394 nonredundant bins with completeness of more than 75% and contamination of less than 25% were obtained. GTDB-tk (v1.1.0) (13) was used to annotate the species of bins, and KofamScan (v1.20) (https://github.com/takaram/kofam_scan) was used to identify the KEGG Orthology with the KEGG Orthology database to find the bins with denitrification genes. Default parameters were used for all software unless otherwise noted.

All of the 16 genomes are *nosZ*-containing genomes, which indicates that there may be potential N_2_O-reducing bacteria (Table 1). Four genomes contain two copies of *nosZ*. There are no nitrate reductase or nitrite reductase genes but having *nosZ* in the genome of DF_1_3.28 implies that this strain could act as an N_2_O sink because it has no capacity to produce N_2_O. The genomes obtained in this study not only enlarge our knowledge of diverse denitrifying bacteria but also facilitate screening of N_2_O-reducing bacteria for use as an N_2_O sink in mitigating greenhouse gas emissions from agricultural environments.

**TABLE 1:** Descriptions and accession numbers of assembled denitrifying bacterial bins

Isolate	Genus/species	GenBank accession no.	Length (bp)	Completeness (%)	Contamination (%)	Coverage (%)	No. of contigs	*N*_50_ (bp)	GC content (%)	No. of denitrification genes					
										*nosZ*	*norB*	*nirK*	*nirS*	*narG*	*napA*
DF_1_1.41	*Flavobacterium* sp.	JAHRWO000000000	2,186,697	75.66	4.79	10.74	402	6,084	32.86	2	1	1	0	0	0
DF_2_2.70	*Lentimicrobium* sp.	JAHRWP000000000	3,205,124	76.79	1.88	17.83	120	43,228	42.75	2	1	1	0	2	0
DR_8_1.35	*Azonexus* sp.	JAHRWQ000000000	3,061,420	81.83	6.81	16.80	455	8,031	62.93	2	1	0	1	0	2
DR_8_1.67	*Thauera* sp.	JAHRWR000000000	3,585,355	77.42	8.18	19.14	569	7,540	69.66	2	0	0	1	1	1
DF_1_1.30	*Diaphorobacter* sp.	JAHRWS000000000	3,271,235	88.97	5.24	16.49	369	11,823	67.49	1	1	0	1	0	0
DF_1_1.72	*Sterolibacterium* sp.	JAHRWT000000000	2,362,309	83.02	3.71	22.18	121	27,074	60.27	1	1	0	0	1	0
DF_1_3.23	Pseudomonas sp.	JAHRWU000000000	3,987,501	82.68	7.28	22.10	583	7,849	64.88	1	3	0	1	1	2
DF_1_3.28	*Cloacibacterium* sp.	JAHRWV000000000	1,902,599	78.95	2.21	10.46	326	7,044	33.03	1	1	0	0	0	0
DF_7_1.31	*Thermomonas* sp.	JAHRWW000000000	2,694,426	98.54	1.71	22.23	56	74,073	58.72	1	2	1	0	1	0
DF_7_2.41	Sphingobacterium mizutaii	JAHRWX000000000	4,230,372	98.89	0	20.58	26	236,025	40.29	1	1	1	0	0	0
DF_8_1.35	*Kaistella* sp.	JAHRWY000000000	2,153,008	87.46	5.64	10.67	287	9,320	39.66	1	0	1	0	0	0
DR_1_1.49	*Castellaniella* sp.	JAHRWZ000000000	3,463,312	91.22	4.34	20.55	102	77,844	63.47	1	2	2	0	1	0
DR_1_3.37	*Rhizobium* sp.	JAHRXA000000000	4,531,742	90.96	8.4	14.72	619	8,744	63.15	1	1	2	0	1	0
DR_7_2.37	*Bdellovibrio* sp.	JAHRXB000000000	2,929,297	90.3	0.18	8.85	317	12,023	46.74	1	1	1	0	1	0
DR_7_3.16	Achromobacter denitrificans	JAHRXC000000000	6,264,563	87.97	1.54	26.88	104	107,223	67.72	1	1	1	0	1	1
DR_8_2.10	*Comamonas* sp.	JAHRXD000000000	2,293,750	75.03	2.74	15.55	286	10,109	64.69	1	0	0	0	0	0

### Data availability.

The raw metagenomic sequence reads and metagenome-assembled genomes were deposited in DDBJ/ENA/GenBank under BioProject accession number PRJNA736218, with BioSample accession numbers SAMN19613211 to SAMN19613226 and SRA accession numbers SRR15558355 to SRR15558366.
